# Two-step strategy for the identification of SARS-CoV-2 variant of concern 202012/01 and other variants with spike deletion H69–V70, France, August to December 2020

**DOI:** 10.2807/1560-7917.ES.2021.26.3.2100008

**Published:** 2021-01-21

**Authors:** Antonin Bal, Gregory Destras, Alexandre Gaymard, Karl Stefic, Julien Marlet, Sébastien Eymieux, Hadrien Regue, Quentin Semanas, Constance d’Aubarede, Geneviève Billaud, Frédéric Laurent, Claudia Gonzalez, Yahia Mekki, Martine Valette, Maude Bouscambert, Catherine Gaudy-Graffin, Bruno Lina, Florence Morfin, Laurence Josset, the COVID-Diagnosis HCL Study Group, COVID-Diagnosis HCL Study Group, COVID-Diagnosis HCL Study Group, Jean-Sébastien Casalegno, Emilie Frobert, Vanessa Escuret, Vinca Icard, Marion Jeannoel, Marie-Paule Milon, Christophe Ramière, Caroline Scholtès, Jean-Claude Tardy, Mary-Anne Trabaud, Isabelle Schuffenecker

**Affiliations:** 1 Laboratoire de Virologie, Institut des Agents Infectieux, Laboratoire associé au Centre National de Référence des virus des infections respiratoires, Hospices Civils de Lyon, Lyon, France; 2 These authors contributed equally to this article; 3 CIRI, Centre International de Recherche en Infectiologie, Team VirPath, Univ Lyon, Inserm,; U1111, Université Claude Bernard Lyon 1, CNRS, UMR5308, ENS de Lyon, Lyon, France; 4 Service de Bactériologie-Virologie-Hygiène, CHRU de Tours, France; INSERM U1259, Université de Tours, Tours, France; 5 Occupational Health and Medicine Department, Hospices Civils de Lyon, Lyon, France; 6 CIRI - Centre International de Recherche en Infectiologie, Team Pathogenesis of staphylococcal infections Inserm U1111, CNRS UMR5308, ENS Lyon, Université Claude Bernard Lyon 1, Lyon, France; 7 Laboratoire de Bactériologie, Institut des Agents Infectieux, Hôpital de la Croix-Rousse, Hospices Civils de Lyon, Lyon, France; 8 The members of the COVID-Diagnosis HCL Study Group are listed below

**Keywords:** SARS-CoV-2, COVID-19, whole genome sequencing, spike protein, variants, deletion

## Abstract

We report the strategy leading to the first detection of variant of concern 202012/01 (VOC) in France (21 December 2020). First, the spike (S) deletion H69–V70 (ΔH69/ΔV70), identified in certain SARS-CoV-2 variants including VOC, is screened for. This deletion is associated with a S-gene target failure (SGTF) in the three-target RT-PCR assay (TaqPath kit). Subsequently, SGTF samples are whole genome sequenced. This approach revealed mutations co-occurring with ΔH69/ΔV70 including S:N501Y in the VOC.

Since September 2020 a severe acute respiratory syndrome coronavirus 2 (SARS-CoV-2) deletion H69–V70 (ΔH69/ΔV70) in the spike (S) protein has attracted increasing attention. This deletion was detected in the cluster-5 variant, identified both in minks and humans in Denmark. This cluster-5 variant carries a receptor binding domain (RBD) mutation Y453F and was associated with reduced susceptibility to neutralising antibodies of sera from recovered coronavirus disease (COVID-19) patients [1-3]. The ΔH69/ΔV70 has also co-occurred with either one of two other noteworthy RBD mutations [4]: N439K that is currently spreading in Europe and might also be related to reduced susceptibility to SARS-CoV-2 antibodies [5] and N501Y that was identified for example in the SARS-CoV-2 variant of concern (VOC) 202012/01 recently detected in England [6]. Although the impact of ΔH69/ΔV70 on SARS-CoV-2 pathogenesis is not clear, enhanced surveillance is urgently needed.

Herein we report the two-step strategy that enabled the timely detection of VOC 202012/01 in France, as well as other variants carrying ΔH69/ΔV70.

## ΔH69/ΔV70 associated with S-gene target failure of a three-target RT-PCR assay

**Figure 1:**
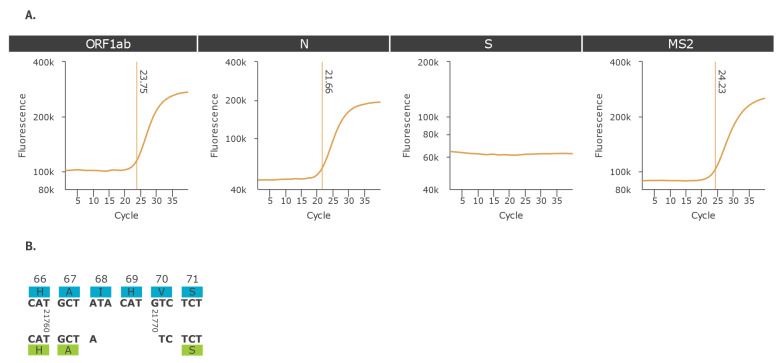
(A) Amplification curves^a^ obtained with TaqPath COVID-19 RT-PCR kit for samples harbouring SARS-CoV-2 with nt deletion 21765–21770 in the spike gene and (B) pairwise sequence alignment from nt position 21758 to 21775 of a reference spike gene and a spike gene with the 21765–21770 deletion

As part of routine SARS-CoV-2 genomic surveillance performed at the national reference centre (NRC) for respiratory viruses (Lyon, France) [7], a 6-nt deletion (21765–21770) within the S gene was identified in two nasopharyngeal samples collected in Lyon, France on 1 and 7 September 2020, respectively. The SARS-CoV-2 infection diagnosis had been performed on 2 and 8 September 2020, respectively with the Applied Biosystems TaqPath RT-PCR COVID-19 kit (Thermo Fisher Scientific, Waltham, United States (US)) that includes the open reading frame (ORF) 1ab, S, and nucleocapsid (N) gene targets. For these two samples, a S-gene target failure (SGTF) was observed while ORF1ab and N targets was correctly amplified with quantification cycle (Cq) values < 25 (Figure 1A).

Cq: cycle quantification cycle; MS2: MS2 bacteriophage internal positive control; N: nucleocapsid gene target; ORF1ab: open reading frame 1ab gene target; S: spike gene (S gene) target; SARS-CoV-2: severe acute respiratory syndrome coronavirus 2.

^a^ The amplification profiles for the three targets (ORF1ab, N, S panels) included in the RT-PCR kit are shown for a SARS-CoV-2 positive sample harbouring the S-gene deletion 21765–21770. The amplification curve of the internal control is also represented (MS2 panel). For each plot, the vertical straight orange line indicates the determination of the Cq, whose value is indicated by a number next to the line.

^b^ Alignment using CoV-GLUE resource [8]. The sequence within the S gene, which lacks nts (i.e. 21765–21770 deletion) is in the lower part of the alignment and the reference sequence (Wuhan-Hu-1) is in the upper part. nt positions in each sequence are indicated by the vertical numbers. Amino acids encoded by the nt sequence containing the deletion are in green boxes, while those encoded by the nt reference sequence are in blue boxes. Amino acid numbering is above the blue boxes. The 21765–21770 deletion in the S gene results in deletion of amino-acid residues 69 and 70 in the S protein; on each side of the 21765–21770 deletion in the nt sequence, the remaining ATC (21764–21771–21772) encodes an isoleucine amino acid (I).

The two samples were subjected to whole genome sequencing (WGS) and the sequences were deposited in the GISAID Initiative’s EpiCOV database on 15 October 2020 (GISAID accession numbers: EPI_ISL_582112, EPI_ISL_582120). The mean coverage was 6903× and 6898×, respectively and the S deletion 21765–21770 was present in 100% of the reads. Using CoV-GLUE online resource [8], we found that the S deletion 21765–21770 led to the removal of two amino acids (ΔH69/ΔV70) in the N-terminal domain of the S1 subunit of the S protein (Figure 1B). The WGS method used was the amplicon-based ARTIC v3 protocol (https://artic.network/ncov-2019) combined with Nextera DNA Flex library and sequencing on NextSeq 500 platform (Illumina, San Diego, US). To confirm the deletion, one sample was also sequenced with an untargeted metagenomic protocol that yielded the same sequence. Of note, this metagenomic approach could not be applied for the second sample due to low viral load [9].

Although the coordinates of the primer/probe binding regions were not available for the TaqPath kit, the manufacturer confirmed that the S deletion H69–V70 was in the area targeted by the test.

## ΔH69/ΔV70 screening with RT-PCR followed by whole genome sequencing

**Figure 2:**
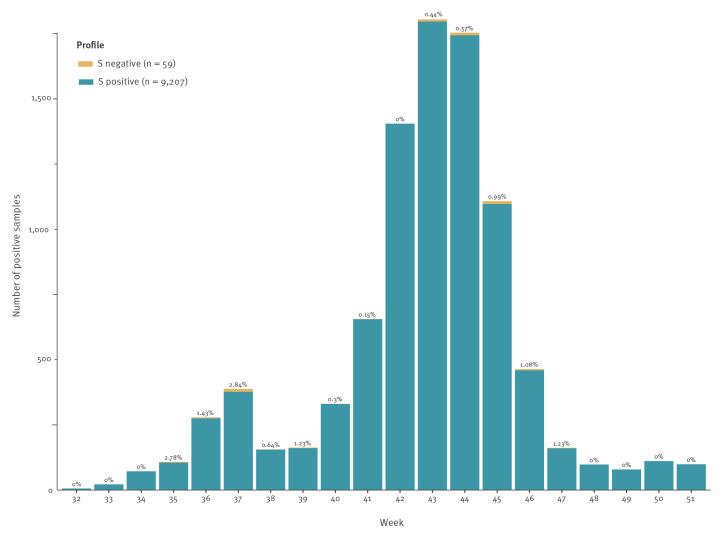
Prevalence of the S negative profile (negative for S target and positive for N and ORF1ab targets) with TaqPath COVID-19 RT-PCR kit, Lyon, France, 3 August (week 32)–20 December (week 51) 2020 (n = 9,266 samples tested)

We then performed a retrospective analysis of the TaqPath kit results obtained at a community-based testing platform hosted by the virology laboratory of Lyon university hospital, from 3 August to 20 December 2020. We selected only the samples with a Cq value < 25 for the N target, the most sensitive target of the test. Of note, a Cq value of 25 with the TaqPath kit corresponds to a cycle threshold (Ct) value of 30 with the real time RT-PCR IP4 (Institut Pasteur assay, Paris, France) [10]. By doing so, we found that 59 of 9,266 (0.6%) positive tests had no amplification of the S gene. No significant increase of the SGTF was noticed over time; the proportion ranging from 0% (week numbers 32, 33, 34, 42, 48–51) to 2.84% (week number 37; Figure 2).

N: nucleocapsid; ORF: open reading frame; S: spike.

Among the 59 samples with SGTF, 36 were available for WGS. These 36 samples were collected from 5 August to 11 November (18/36 were collected after 9 October). A total of 11 samples that presented an amplification of the S target were also sequenced. The sequencing results were fully concordant with the RT-PCR profiles (all of the samples with SGTF had the S deletion ΔH69/ΔV70, while all of the S-gene positive samples did not contain ΔH69/ΔV70). For samples with SGTF, other S mutations were detected and are summarised in the Table. For 21 of the 36 samples, the S mutations co-occurring with ΔH69/ΔV70 were S477N and D614G; 10 of the 36 samples presented N439K and D614G mutations in addition to ΔH69/ΔV70. Of note, the complete combination of S mutations in cluster-5 variant or in VOC 202012/01 was not found in any of the 36 samples.

**Table:** Spike mutations co-occurring with ΔH69/ΔV70 in samples with S negative profiles (negative for S target and positive for N and ORF1ab targets) obtained with the RT-PCR TaqPath kit, Lyon, France, 5 August–11 November 2020 (n = 36 samples)

Spike mutation co-occurring with ΔH69/ΔV70 spike deletion	Number of sequences
S477N + D614G	21
N439K + D614G	10
H146Y + D614G	1
D80Y + N439K + D614G	1
ΔI670/Δ671/Δ672/Δ673 deletion + S477N + D614G	1
D614G	1
V401L + S477N + D614G	1

N: nucleocapsid; ORF: open reading frame; S: spike.

The two-step strategy (i.e. screening with the TaqPath COVID-19 RT-PCR kit followed by WGS for SGTF samples) presented herein has been implemented in France since 20 December 2020. On 21 December, the virology laboratory of university hospital of Tours reported a SGTF on a nasopharyngeal sample from a patient with a recent travel history from England (London). The sample was sent to the NRC for WGS and the detection of VOC 202012/01 (lineage B.1.1.7) was confirmed on 25 December, corresponding to the first detection of this variant in France (GISAID accession number: EPI_ISL_735391).

## Ethical statement

Samples used in this study were collected as part of an approved ongoing surveillance conducted by the NRC for respiratory viruses in Lyon, France (World Health Organization reference laboratory providing confirmatory testing for COVID-19). The investigations were carried out in accordance with the General Data Protection Regulation (Regulation (EU) 2016/679 and Directive 95/46/EC) and the French data protection law (Law 78–17 on 06/01/1978 and Décret 2019–536 on 29/05/2019. No additional samples for the purpose of this study were collected. Patients were informed of the research and their non-objection approval was confirmed. This study was presented by the ethics committee of the Hospices Civils de Lyon (HCL), Lyon, France and registered on the HCL database of RIPHN studies (AGORA N°41).

## Discussion and conclusion

According to CoV-GLUE resource [8] (update from GISAID: 14 December 2020), the S deletion 21765–21770 has been identified in 4,632 sequences worldwide (> 99% in Europe). Interestingly, only 16 sequences containing this deletion were sampled between 15 March and 23 July corresponding to the first wave of COVID-19 pandemic in Europe. Herein, using data obtained with TaqPath RT-PCR kit, we found an overall prevalence of 0.6%, suggesting a limited circulation of variants presenting ΔH69/ΔV70 during the second wave of the pandemic in Lyon, France. Of note, at the time of the study no travel restriction measures were implemented in France. The two-step strategy presented herein enabled to identify SARS-CoV-2 variants carrying ΔH69/ΔV70 and RBD mutations. More importantly, this strategy allowed the first detection of the VOC 202012/01 in France.

It should be underlined that N439K, Y453F or N501Y RBD mutations that can co-occur with ΔH69/ΔV70 might be associated with an increased affinity to angiotensin-converting enzyme 2 (ACE2) or reduced sensitivity to SARS-CoV-2 antibodies [3,5,11-13]. The N501Y mutation of the VOC 202012/01, in particular, might be also responsible of the higher transmissibility reported for this lineage [14].

It has been hypothesised that ΔH69/ΔV70 might compensate some RBD mutations and might be involved in the transmissibility of the variants containing these mutations [4,6]. In addition, it has been recently shown that the combined ΔH69/V70 and D796H mutant was less sensitive to neutralising antibodies [15]. As the N-terminal domain of the S1 subunit of the S protein may interact with lung receptors [16] and might be a target of neutralising antibodies [17,18], further studies are needed to understand the consequences of ΔH69/ΔV70 on SARS-CoV-2 transmissibility and host-immune response.

The present study has several limitations. First we selected only samples with a Cq value <25 for the N gene that corresponds to the limit of sensitivity of the WGS method used. The detection of VOC 202012/01 could therefore be underestimated, but not the prevalence. In addition, this strategy cannot identify other variants of concern that do not carry ΔH69/ΔV70 as the variant 501Y.V2 first detected in South Africa [19]. However, it allows rapid retrospective and prospective evaluation of the prevalence of VOC 202012/01, including in countries with limited sequencing capacity.

Importantly, the TaqPath kit did not lead to false negative conclusions regarding the SARS-CoV-2 diagnosis as the two other targets remained positive. The data presented herein emphasise that the TaqPath RT-PCR assay is a useful and cost-effective tool enabling a rapid, large-scale screening of SARS-CoV-2 variants with ΔH69/ΔV70. In the meantime, a similar approach has been adopted in the United Kingdom where the frequency of SGTF is used as a proxy for estimating and monitoring the spread of the VOC 202012/01 [14,20]. Samples with SGTF should be further addressed to national referral laboratories for SARS-CoV-2 WGS in order to confirm the presence of VOC 202012/01. This two-step strategy can contribute to the timely detection and isolation of VOC 202012/01 cases and has been reinforced in France as national diagnostic platforms have mainly implemented the TaqPath RT-PCR kit.
